# Unconventional animal models: a booster for new advances in host—pathogen interactions

**DOI:** 10.3389/fcimb.2014.00142

**Published:** 2014-10-08

**Authors:** Filippo Conti, Prasad Abnave, Eric Ghigo

**Affiliations:** Centre National de la Recherche Scientifique UMR 7278, IRD198, Institut National de la Santé et de la Recherche Médicale U1095, Aix-Marseille UniversitéMarseille, France

**Keywords:** model organism, dictyostelium, planarians, tetrahymena, host-pathogen interactions

## Animals as tools

Historically, biology has greatly benefited and is still benefiting from the study of animals. A quick search of the PubMed database for “animal models” yields some 41,000 studies, and many others will come. Once regarded as the subject of biological studies [Darwin's finches are a well-known example (Peterson, 2008)], animals have become tools to seize and solve the genetic secrets that underlie most of the biological aspects of life. Like in developmental or cancer biology, studies of bacterial infections and more general studies on the interactions involved in host-parasite relationships have been greatly boosted by the availability of animal models that can recapitulate such complex events.

Looking backward for the root of this transition, the work of the English scientist Edward Jenner can be observed as an important turning point. At the beginning of the 19th century, it was known that milkmaids were generally immune to smallpox. Thus, Jenner postulated that the pus that was discharged from the blisters that milkmaids received from cowpox (a less virulent disease) protected them from smallpox. A short time later, Jenner scratched some matter from fresh human cowpox and injected it into a child's arm in an attempt to make him ill with cowpox and, at the same time, to test its protective properties against smallpox. The child became mildly ill with cowpox, fully recovered a week later, and did not contract smallpox once Jenner deliberately inoculated the virus into his arm. The child was then immune, and Jenner went on to test his idea in other humans. Jenner had turned animals (in this case, the cow) into tools, similar to Pasteur, who discovered the vaccines for chicken cholera and rabies a few decades later.

Thereafter, the status of animals changed. They were no longer only subjects of study, they became tools in human hands and, like every tool, animals, such as mice, rabbits and flies, underwent several rounds of optimization. Although experimental immunology began with large animals, today, mouse models, and to a lesser extent *Drosophila* and *C. elegans*, dominate modern biological research. Too often however, scientists think of these models as perfect models of human biology, as if manipulating some genes could actually recapitulate physiology and diseases in different species. Experiment involving mice, genetically modified or not, are extremely helpful to mirror the pathophysiology of most of the diseases, but the comparison human equal mice can turn particularly dangerous. Furthermore, mice used in research are usually young, while many of the diseases that are studied by researchers (such as cancer and neurological diseases) are most common in old people.

Very recently, a large scale collaborative research project showed that inflammation response in human is not depicted by the corresponding mouse models as, the authors stated, “these results show that the genomic responses to different acute inflammatory stresses are highly similar in humans, but these responses are not reproduced in the current mouse models. New approaches need to be explored to improve the ways that human diseases are studied” (Seok et al., 2013).

Moreover, animal research focusing excessively on one laboratory species (mice) may lessen the chance of large scientific advances occurring in the next years.

Here, we will discuss how these chances may be improved greatly by evaluating the unique opportunities that are offered by other unconventional model organisms.

## The revenge of the fallen: dictyostelium, zebrafish, and large animals

**Figure 1:**
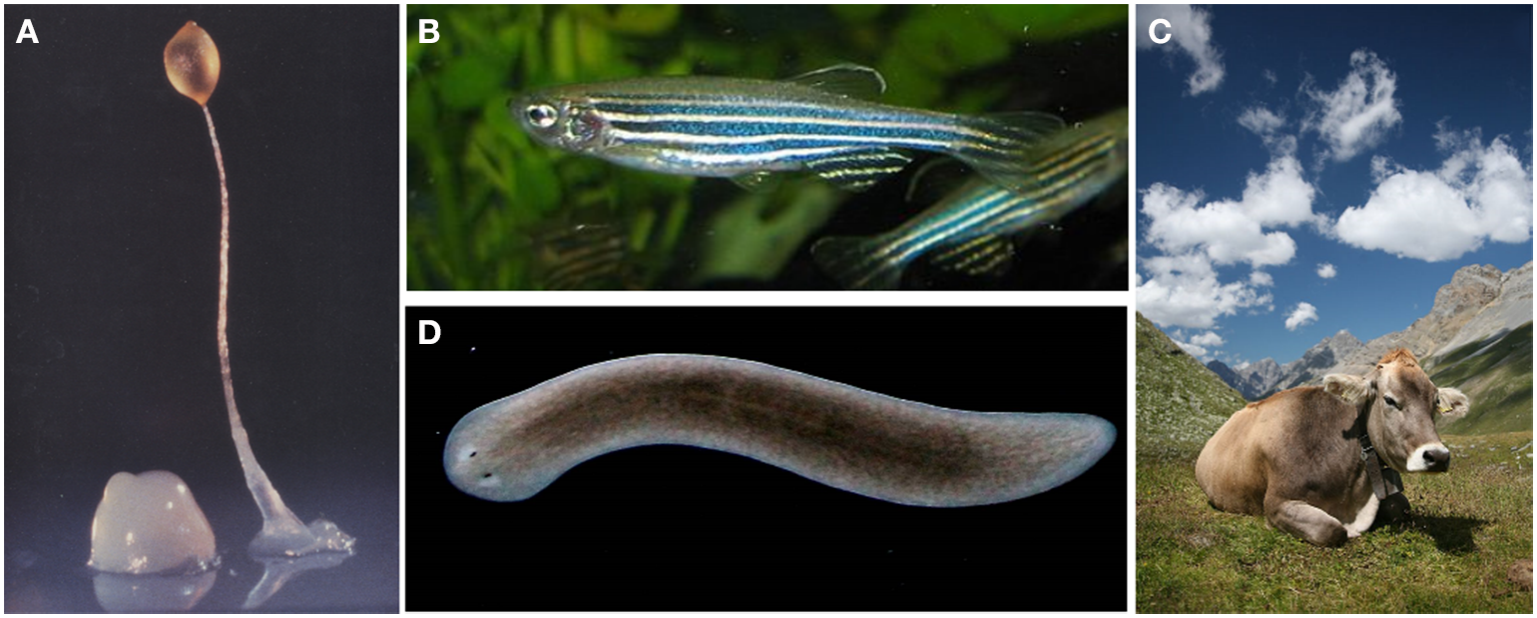
**(A)** The soil-living amoeba *Dictyostelium discoideum*. **(B)** The tropical freshwater fish *Danio rerio* (Zebrafish) **(C)** *Bos taurus*, also known as cow (credit: Wikipedia). **(D)** The freshwater flatworm *Schmidtea mediterranea*. **(A–C)** illustrations are under the Creative Common Attribution License (Credit: Wikipedia).

Living within the soil, *D. discoideum* (Figure 1A) phagocytose bacteria for nutritional purposes. In turn, bacteria have evolved several mechanisms to escape amoeboid phagocytosis, and this defense would add an effective advantage to the bacterium. On the other hand, *D. discoideum* is not without defense against infection by bacteria and has evolved mechanisms to efficiently detect and kill bacteria.

A recent study shows that *D. discoideum* cells can discriminate between Gram-negative and Gram-positive bacteria (Nasser et al., 2013; Snyder, 2013). It is still not clear whether this recognition plays a role in defense against potential pathogenic bacteria, and it is conceivable that metabolic alterations induced in pathways may act as triggers for other defense responses. The group of Adam Kuspa identified different sets of genes that are critical for the survival of *D. discoideum* during feeding on Gram-positive or Gram-negative bacteria. The group showed that the cell-surface glycol-protein gp130, among others, is required for growth on Gram-positive bacteria, whereas the putative AX4 family lysozyme-like protein AlyL is essential for sustained growth on Gram-negative bacteria. Moreover, the same group has shown that the metabolic flux of hexose monomers, from the catabolic breakdown of bacterial cell walls to the anabolic production of pentose monomers, is used by *D. discoideum* to tune the appropriate responses to Gram-positive bacteria (Nasser et al., 2013). Interestingly, a recent study links the burst of pentose phosphate to the activation of macrophages. The sedoheptulose kinase CARKL (Carbohydrate Kinase-Like protein) orchestrates the balance between pro- and anti-inflammatory immune responses through metabolic control. These examples highlight how the study of a very simple amoeba may shed light into the function of innate immune systems in a variety of different organisms (Haschemi et al., 2012).

The expansion of *Danio rerio* (zebrafish) (Figure 1B) organism in biomedical research is establishing it as a suitable disease model to study infection related pathologies such as tubercolosis (TB). In fact, *D. rerio* is naturally susceptible to TB caused by *Mycobacterium marinum* (Mm) and, similarly to mammals, both innate and adaptive immunity are involved in protection against TB infection. In 2002, Davis et al., thanks to the optical transparency of zebrafish embryos, performed a real time visualization of granuloma formation following *M. marinum* infection (Davis et al., 2002). In this study the authors showed that granuloma structures appeared surprisingly at the early step of the infection in a context of innate immunity. This result was remarkable since previous reports suggested that components of adaptive immunity, principally T lymphocytes, played a leading role in the recruitment and activation of macrophages to form Mycobacterium granulomas (Flynn and Chan, 2001).

The use of large animals as experimental models has provided important advances in an increasing number of developmental immunology studies, and swine, horses, cattle, sheep, and deer might be as good as or better than mice for studying several human pathologies such as influenza, tuberculosis, Crohn's disease, asthma, and viral diarrhea. Beyond the obvious advantages due to their size (sampling tissues or liquids and easier surgical intervention), it is important to note that large animals and humans have often developed as out-bred populations over millennia, so it is plausible that their immune systems have been modeled by exposure to a similar extent of infectious agents.

A classic example of convergent disease, even if still controversial, is Crohn's disease in humans, which shows some similarities with Johne's disease in large animals (Figure 1C) (Shanahan, 2002). Johne's disease is caused by *Mycobacterium avium* (*Spp. paratuberculosis* or *MAP*), and the main clinical signs, which are rarely evident until two or more years after the initial infection, are diarrhea and wasting. Several studies showed that a high percentage of people with Crohn's disease are infected with *M. avium* (*Spp. paratuberculosis*). Interestingly, recent studies have shown that IL-23 plays a central role in driving the inflammatory response in Crohn's disease; therefore, extending these studies to cattle at the clinical stage of infection might reveal that IL-23 also plays a central role in Johne's disease and that IL-23 might be one of the factors involved in the breakdown in protective immunity. Despite *M. paratuberculosis* began first proposed as an etiologic agent in Crohn's disease more than 25 years ago (Davis and Madsen-Bouterse, 2013), in some cases, there is no clear evidence indicating that *M. avium* is a causative agent or that its presence only represents an incidental association. More detailed studies in cattle may provide background information for comparisons to the immune response during the latent stage of MAP infection in healthy subjects and in patients with Crohn's disease.

## From here to eternity: the planarians experience

Planarians (Figure 1D) are non-parasitic flatworms that live in fresh waters. They are mainly known by the scientific community for their ability to almost limitlessly regenerate thanks to the high presence of neoblast throughout their tissues. Neoblast are pluripotent somatic stem cells present in the parenchyma and they can give rise to all other 30–40 different cell types. Due to this property, planarians have been extensively used as an animal model: they are cheap, small and can multiply by simply cutting their body, which is a property that is mainly used for developmental biology studies. Finally, they do not rise any ethical concern (Newmark and Sánchez Alvarado, 2002; Sánchez Alvarado, 2007).

Lately, planarians have become one of the model references for studying stem cell biology, but these flatworms may also be useful for studying other biological issues. More than 20 years ago, M. Morita and T. Sakurai (Ishii and Sakurai, 1991; Morita, 1991; Morita and Collins, 1995) noted the peculiar role of certain cells called “reticular cells” that could mediate the early immune response. These high-degree mobility cells could recognize foreign material, such as bacteria, as early as 8 h after their introduction. In 2012, Zhou et al. characterized a serine protease whose expression was induced after ingestion of bacteria (a non-pathogenic laboratory strain of *E. coli DH5*α) in the flatworm *Dugesia japonica* (Zhou et al., 2012). As serine proteases may be mediators of immune responses in mosquito, the authors hypothesized that induction, which is specifically triggered when the worms were challenged with bacteria, could represent a first step of a wider and unknown host-pathogen relationship in planarian.

Finally, it has been shown (Abnave et al., 2014) that planarians are highly resistant to infections by bacteria that are highly pathogenic to humans, *C. elegans* and *D. melanogaster*, and planarian display a genuine immune response involving at least three genes with orthologs in humans, MORN2 (Membrane Occupation and Recognition Nexus-2 protein), DUSP19 (Dual-Specificity phosphatase enzyme), and PAQR3 (progestin/adipoQ receptor-3). Particularly, they demonstrated for the first time that MORN2 has a role in LC3-associated phagocytosis (LAP), and it is essential in eliminating bacterial pathogens by human macrophages as well as by flatworms. Thus, the dataset collected by P. Abnave and colleagues highlights, for the first time, a major interest in studying planarian defense mechanisms to identify conserved immune factors.

Last year, the discovery that *S. mansoni*, a parasitic tapeworm that is the cause of Schistosomiasis, one of the most prevalent human parasitic diseases, has its own neoblast population prompted researchers to hypothesize that some species of planarians (*Schmidtea mediterranea* and *D. japonica*) might be good models for studying the disease, whose cure currently relies on a single compound, praziquantel (Collins and Newmark, 2013; Collins et al., 2013).

## Think differently

It is now widely accepted that mouse models, although still very precious, may show some limits in simulating human biological processes or diseases (Seok et al., 2013; Özdemir et al., 2014). Using non-human primates may be a straightforward solution because their physiologies are closer to that of humans, but economical and ethical issues are a barrier for most research institutes. Regarding host-parasite diseases, working on new model such zebrafish might open completely new routes of study. In the same way, using *S. mediterranea* or *D. japonica* flatworms as model organisms (actually absent) to study the physiology of the parasite *S. mansoni* might help us to find new drugs to counter Schistosomiasis, whose control currently relies on a single drug. On the other hand, some less-considered animals could effectively mirror the human immune response. Cow, for example, can be a suitable model to study intestinal and uterine infection because the production of lipopolysaccharides or pro-inflammatory cytokines is similar to that observed during human inflammation processes.

It is indisputable that mouse models are and will be for a long time the most suitable and convenient animal model in biological research, but it is now clear that mice do not represent the best biological model due to the intrinsic differences between rodents and humans. Hence, there is interest in developing and studying less regarded organisms that, as the few examples here have shown, may be greatly useful to the biological understanding of complex host-pathogen interactions.

### Conflict of interest statement

The authors declare that the research was conducted in the absence of any commercial or financial relationships that could be construed as a potential conflict of interest.
